# Analysis of the efficacy of microdissection of paravebous sinus meningiomas invading large venous sinuses

**DOI:** 10.1186/s12893-023-01999-4

**Published:** 2023-05-06

**Authors:** Chengyuan Ji, Jiashuo Zhao, Weixin Xing, Jiangang Liu

**Affiliations:** Department of Neurosurgery, The First Affiliated Hospital of Soochow University, No.188 Shizi Street, Suzhou, 215000 Jiangsu China

**Keywords:** Paravebous sinus meningiomas, Venous sinus, Venous sinus reconstruction, Tumor recurrence

## Abstract

**Objective:**

The management of paravebous sinus meningiomas that invade major venous sinuses is a subject of debate, particularly concerning the necessity of complete resection of the tumor and reconstruction of the venous sinus. This article aims to demonstrate the outcomes of total removal of the lesion (including the invading venous sinus portion) and the effects of restoring or not restoring venous circulation in terms of recurrence of the tumor, mortality, and post-operative complications.

**Methods:**

The authors conducted a study involving 68 patients with paravebous sinus meningiomas. Of the 60 parasagittal meningiomas, 23 were located in the anterior third, 30 in the middle third, and 7 in the posterior third. Additionally, 3 lesions were located in the sinus confluence area, and 5 in the transverse sinus. All patients underwent surgery, and the degree of venous sinus involvement was classified into six types. For type I meningiomas, the outer layer of the sinus wall was stripped off. For types II to VI, two strategies were employed: non-constitutional, wherein the tumor and affected venous sinuses were removed without repair, and reconstructive, wherein the tumor was completely removed and the venous sinuses were sutured or repaired. Karnofsky Performance Status (KPS) scale and Magnetic Resonance Venography (MRV) were utilized to assess the outcomes of the surgical procedures.

**Results:**

The study group of 68 patients underwent complete tumor resection in 97.1%, with sinus reconstruction attempted in 84.4% of cases with sinus wall and sinus cavity invasion. The recurrence rate of this group was 5.9%, with follow-up ranging from 33 to 57 months. It was found that the recurrence rate was significantly higher in cases with incomplete resection than in those with complete resection. The overall mortality rate was 4.4%, with all cases resulting from malignant brain swelling due to the failure to perform venous reconstruction after resectioning of the meningioma type VI. Furthermore, 10.3% of patients experienced worsening symptoms of neurological deficits or complete loss of neurological function, with a significantly higher incidence in those without venous reconstruction than in the venous reconstruction group (*P* < 0.0001, Fisher test). No statistically significant pre-operative and post-operative KPS differences were observed in patients with type I to V. However, in patients with type VI (who did not receive venous reconstruction), the post-operative KPS score was significantly worse.

**Conclusion:**

The results of this study suggest the necessity of a complete resection of the tumor, including the invasive venous sinus component, as the recurrence rate was found to be relatively low at 5.9%. Moreover, patients who did not undergo venous reconstruction showed significant deterioration in their clinical condition compared to other subgroups, thus highlighting the importance of venous sinus reconstruction.

**Supplementary Information:**

The online version contains supplementary material available at 10.1186/s12893-023-01999-4.

## Background

Microsurgical resection of parasagittal meningiomas is still a complicated process, which can have lasting neurological consequences, especially when the superior sagittal sinus is invaded [1]. Reports have suggested that radiation therapy is effective for tumors that remain in the venous sinus wall or within the venous sinus [1, 2]. However, the recurrence rate of tumors after radiation therapy has been estimated to be 18-22% [3], indicating that radiation therapy is inaccurate in reducing the probability of recurrence of paranasal meningioma. The conundrum of whether to actively perform surgery on the portion of the paravenous sinus meningioma that has infiltrated the venous sinus, along with reconstructing the sinus wall post-resection, is a contentious issue.

Taking a more aggressive approach, some medical centers practice the removal of the tumor outside the venous sinus, followed by electrocautery of the sinus wall or any residual tumor tissue within the sinus and post-operative supplemental radiation therapy. This results in a better prognosis for the patient than the removal of the tumor and the invaded venous sinus alone [1]. It has been reported that the recurrence rate of meningioma is 6% when the tumor is completely removed and the dural attachment is also taken out. However, if only the tumor is completely removed and the attachment part is treated with electrocoagulation cautery, the recurrence rate increases to 16%. The recurrence rate is highest, at 29%, when the tumor is simply resected and the attachment part is not treated at all, suggesting that the removal of the attachment part of the tumor can effectively reduce the recurrence rate and improve the prognosis [3].

In cases where the venous sinus is not completely occluded, resection of the involved sinus requires sinus repair to ensure proper venous return. However, when the venous sinus is wholly blocked, it has been suggested that a one-stage reconstruction should be respected, rather than resecting the occluded sinus without reconstruction. It has been reported that when the venous sinus is wholly occluded, there is often a compensatory intracranial collateral venous circulation, thus negating the need for reconstruction [4–7]. Nevertheless, recent studies conducted by Sindou et al. have demonstrated that in paranasal meningiomas with complete venous sinus occlusion, patients with venous sinus reconstruction have a better prognosis [5, 8].

We conducted a retrospective study of 68 patients who underwent surgical treatment for paravebous sinus meningiomas at our neurosurgery department between 2017 and 2019 to evaluate which surgical approach was most beneficial for the prognosis of patients with paranasal meningioma. Our investigation included comparing the recurrence rates, complications, mortality, and KPS scores among patients with different surgical approaches.

## Methods

### Characteristics of the patients

This study included 68 patients who underwent surgery in a neurosurgery department between 2017 and 2019. All surgeons were experienced in their field. The patients were monitored for 33 to 57 months on average (44.87 ± 7.13 months). Of the 68 patients, 14 were male and 54 were female, with ages ranging from 32 to 82 years (58.71 ± 10.6 years). It was determined that 60 of them had tumors located in the superior sagittal sinus parietal: 23 in the anterior third, near the anterior central vein, 30 in the middle third, close to the posterior central vein, and 7 in the posterior third. Additionally, 3 lesions were located in the sinus confluence area and 5 in the transverse sinus area. The inclusion and exclusion criteria are outlined in the Supplementary material. Computed Tomography (CT) scans and Magnetic Resonance plus Magnetic Resonance Venography (MR & MRV) imaging were performed to evaluate the size, location, and extent to which the tumor had invaded the venous sinus cavity.

**Fig. 1:**
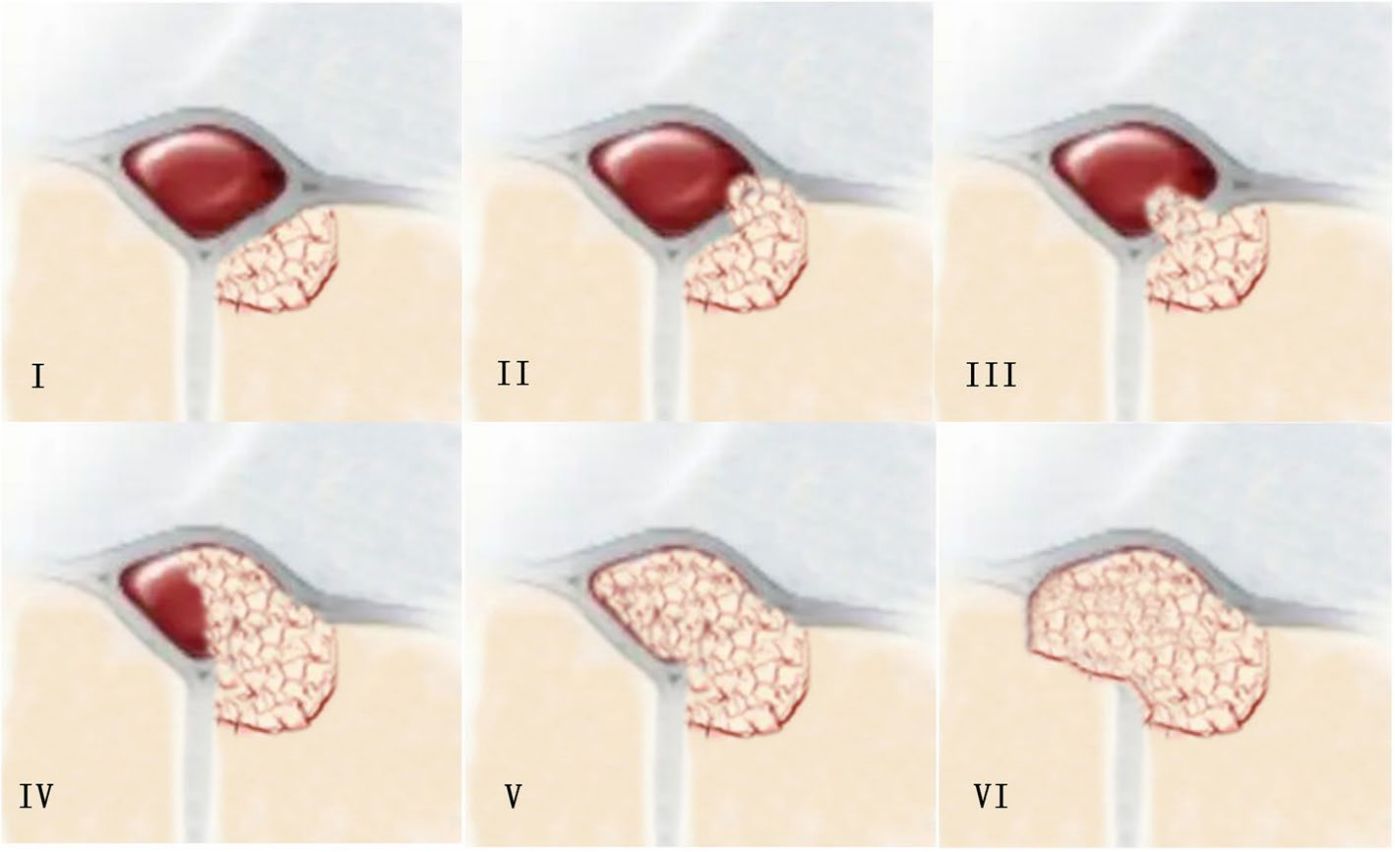
Classification of meningiomas according to the type of invasion of the sinus: type I, meningioma attached to the external surface of the sinus wall; type II, lateral recess invaded; type III, ipsilateral wall invaded; type IV, both ipsilateral wall and sinus apex invaded; type V, sinus completely occluded but contralateral wall not invaded; type VI, sinus completely invaded and contralateral wall invaded

The WHO classification system was used to histopathologically stage meningiomas in our study (Table 1). Among the 60 patients with parasagittal meningiomas, 21 were classified as type I invasive, 8 as type II, 12 as type III, 11 as type IV, 4 as type V, and 5 as type VI. Of the 3 patients with meningioma of the sinus confluence, 1 was type IV, 1 was type V, and 1 was type VI. Of the 5 patients with transverse sinus meningiomas, 2 were type I, 1 was type III, and 2 were type IV (Table 2). This classification scheme (Fig. 1) was based on the venous sinus invasion system [8, 9].

**Table 1:** Histopathological of paravebous sinus meningiomas invading the dural sinuses according to the WHO classification

Histopathological typing	No. of Cases
fibrous	27
transitional	21
meningothelial	13
atypical	5
clear cell type	2
total	68

**Table 2:** Distribution of meningioma cases

Location	Sinus typing	No. of Cases
Parasagittal meningioma (60 cases)	I	21
	II	8
	III	12
	IV	11
	V	4
	VI	4
Meningioma of sinus confluence area (3 cases)	IV	1
	V	1
	VI	1
transverse sinus meningioma (5 cases)	I	2
	III	1
	IV	2

### Surgical procedure

The treatment for sinus invasion depends on the type. The first option involves resection of the tumor and then resection of the outer dural layer followed by electrocoagulation of the inner dural layer at the tumor attachment. The second option is to resect the tumor, remove the invading sinus wall, and reconstruct the sinus wall via direct suture or patch repair. The third option is to excise the tumor along with the attached part of the venous sinus but without reconstruction of the venous sinus. Complete resection, as defined by Simpson I or II level resection, and total resection except for small electrocoagulation cautery residues (Simpson III level resection), was confirmed three months post-surgery through enhanced CT or enhanced MR examination, with no recurrence or residual tumor present [3].

**Fig. 2:**
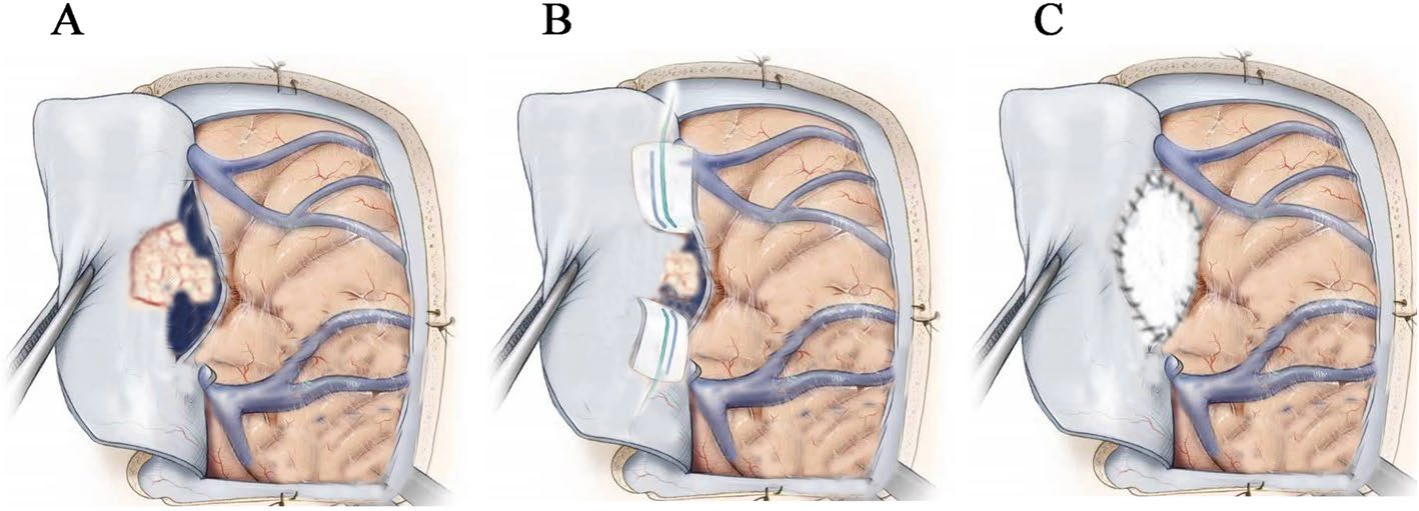
Schematic diagram of sinus repair for type IV meningioma: **A** The venous sinus cavity is opened to expose the tumor tissue in the sinus. **B** Control of venous bleeding using gelatin sponge + tape cotton. **C** Venous sinus reconstruction using an artificial patch

The position of the patient is determined by the location of the tumor, with the exception of tumors in the first third of the upper sagittal sinus, which requires head fixation during surgery. The incision and bone window exposure (Fig. 2) should be sufficient to expose the tumor and the venous sinus, with visibility on both sides of the sinus and approximately 3 cm proximal and distal to the edge of the occluded sinus as described before [8]. During the sinus wall incision, compression with a gelatin sponge and cotton tape was employed to stop the bleeding; thereafter, the venous sinus tumor was removed, without the need to use aneurysm clips to control the bleeding. Depending on the circumstances, direct closure of the sinus wall with 8 − 0 Prolene sutures or repairs with patches were performed. In patients who have undergone venous sinus repair with a patch, a head CT scan is conducted 24 h post-surgery to ensure that no intracranial hematoma has occurred. Prophylactic anticoagulation is administered for two weeks, with daily subcutaneous injections of 0.3 mL of low-molecular-weight heparin calcium injection. During this period, fibrinogen, bleeding time, coagulation time, prothrombin time, thrombin time, activated partial thromboplastin time (APTT) and platelet levels are monitored. If the APTT value is more than twice the average, anticoagulation therapy should be discontinued. Furthermore, the skin and mucous membranes of the entire body should be carefully examined for any signs of bleeding [10].

### Follow up

Upon the patient’s discharge, a repeat cranial MR & MRV was performed to verify the venous sinuses’ patency, and another MR & MRV was conducted at three months postoperatively to look for tumor remnants and recurrence at three years postoperatively. Afterward, only clinical follow-up evaluations were conducted, and imaging was only conducted when new clinical manifestations arose. Patients’ functional status was assessed using the Karnofsky Performance Status (KPS) scale, and any mortality within three months post-surgery was deemed as related to the procedure. To ensure no new clinical manifestations occurred, patients were monitored through telephone inquiry or outpatient review, with neurological morbidity defined as any worsening of a previous neurological deficit or the emergence of a new deficit that persisted until the third post-operative month.

### Statistical methods

Graphpad Prism 8.3 was utilized for statistical analysis, with the measurement data expressed as mean ± standard deviation and the count data expressed as rates. ANOVA tests were utilized to assess the variations in KPS scores between the surgical groups for continuous numerical data. Non-parametric tests was employed to analyze the differences in pre-operative and post-operative KPS scores within each surgical group or between two groups. Fisher’s analysis and chi-square test were used to investigate the disparities in recurrence and mortality rates between two or three groups. The means were expressed with 95% confidence intervals. Results with a *P*-value of less than 0.05 were considered statistically significant.

## Results

### Details of the surgical procedure

**Fig. 3:**
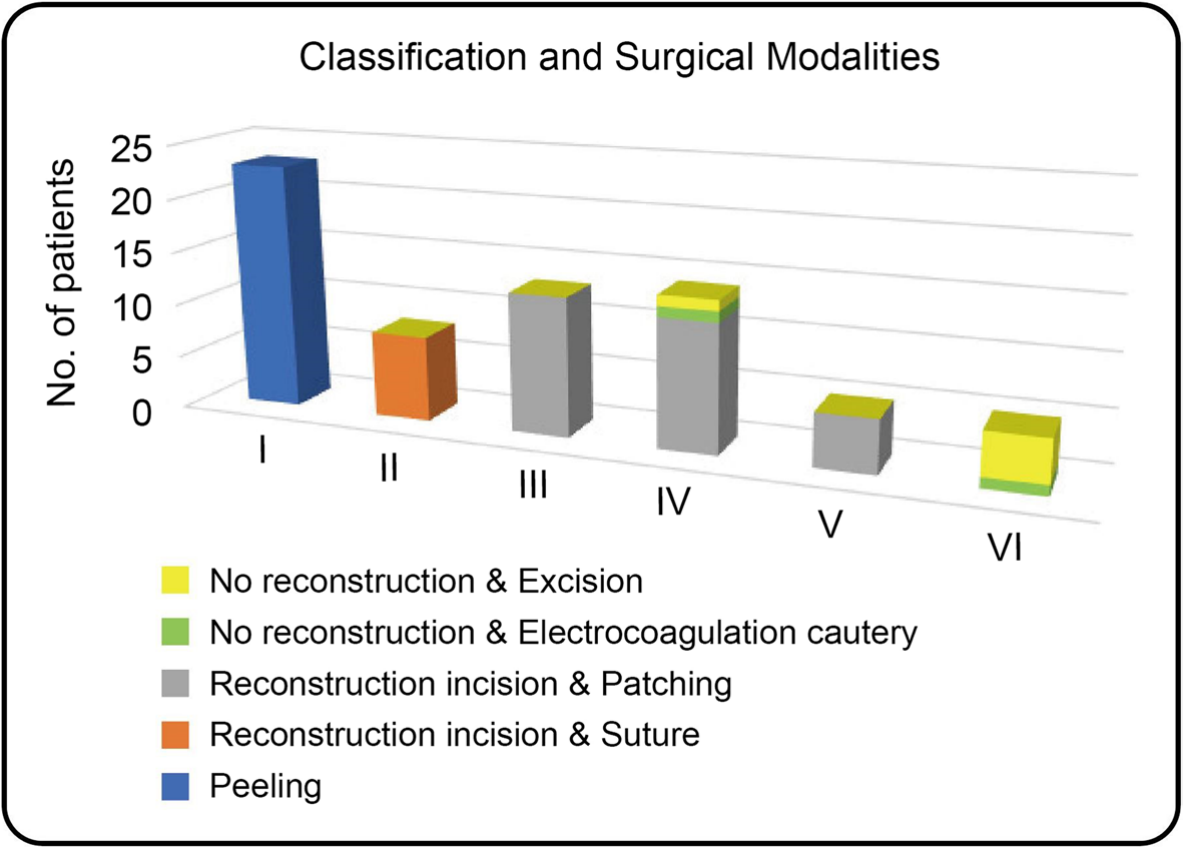
Determination of surgical approach based on sinus tract invasion classification

**Fig. 4:**
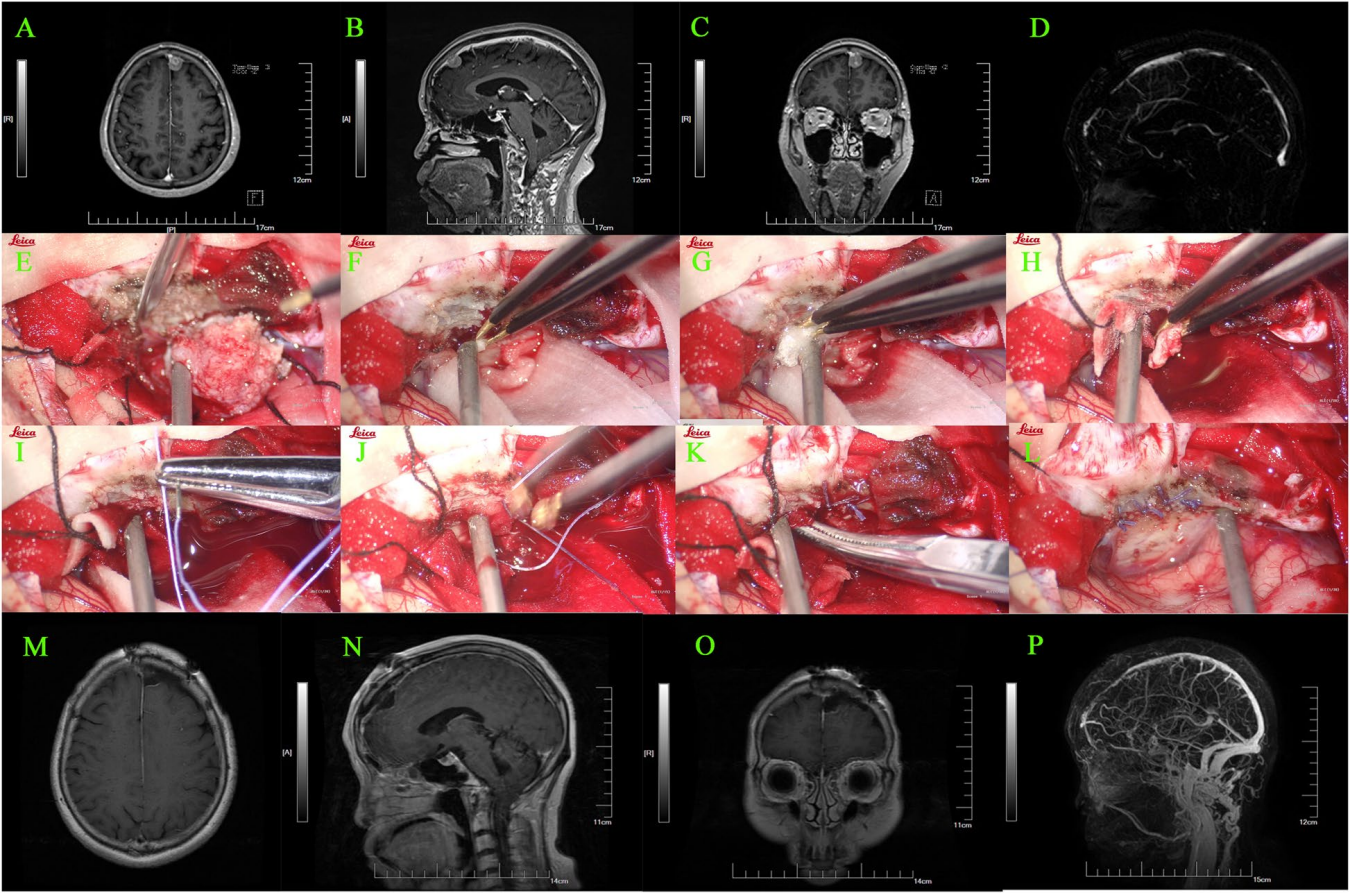
** A-C** Type II paravebous sinus meningioma in the anterior third of the superior sagittal sinus. **D** compression thinning of the anterior third of the superior sagittal; **E-L** complete resection of the tumor and removal of the tumor from the wall of the sagittal sinus, followed by suturing of the sinus wall; **M-O** post-operative MR suggested complete resection of the tumor; **P** post-operative MRV suggesting patency of the venous sinus

**Fig. 5:**
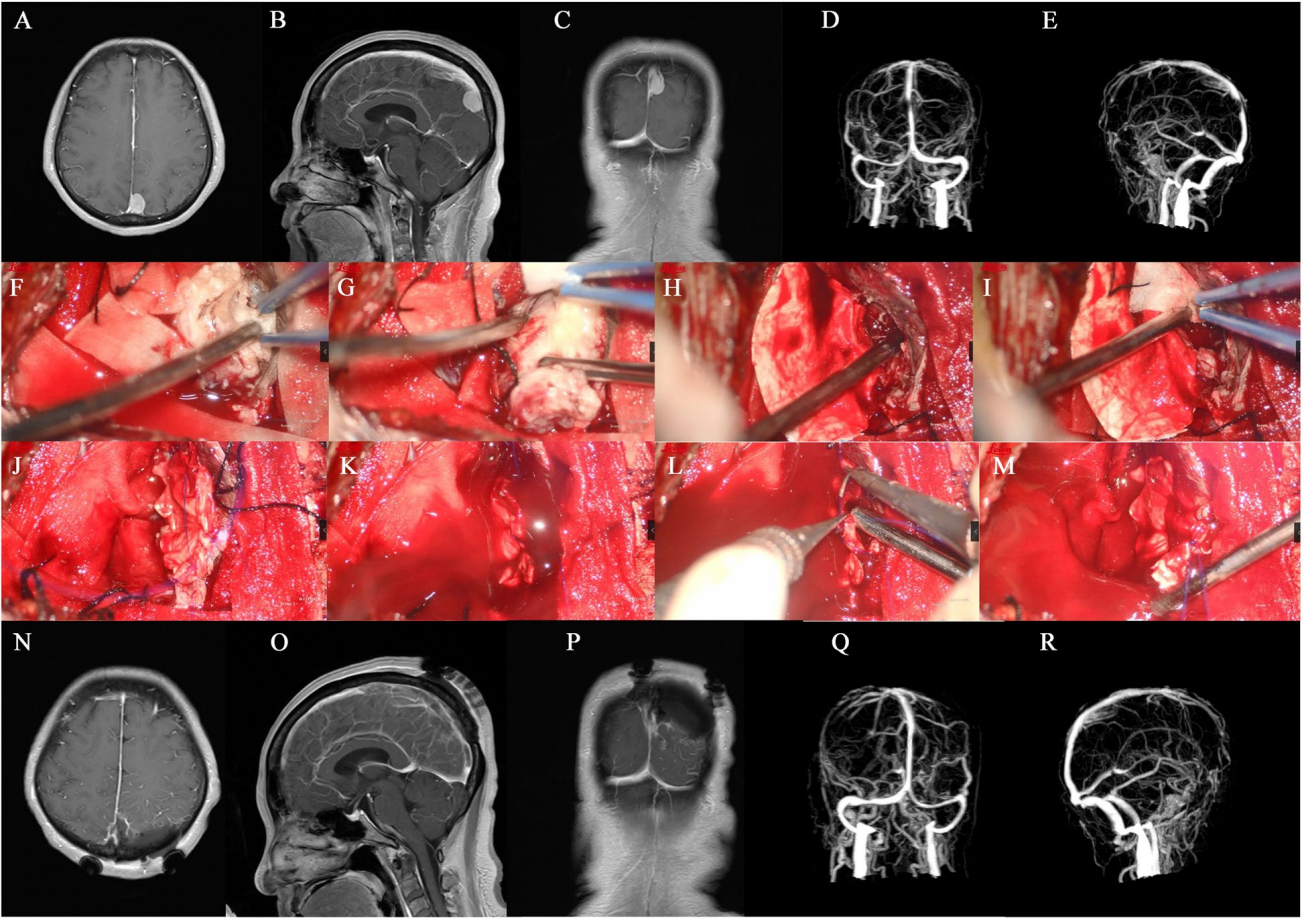
** A-E** pre-operative MR and venous sinus of the patient with type IV. The patient’s tumor was located in the posterior third of the superior sagittal sinus, and the tumor invaded the ipsilateral wall and top of the superior sagittal sinus, and MRV imaging showed significant narrowing of the venous sinus; **F, G** resection of the extra-sinus tumor portion; **H, I** opening of the venous sinus and compression with gelatin sponge + cotton tape to stop bleeding; **J-M** patch suture of the venous sinus; **N-R** complete resection of the tumor, and post-operative MRV indicated patency of the venous sinus

**Fig. 6:**
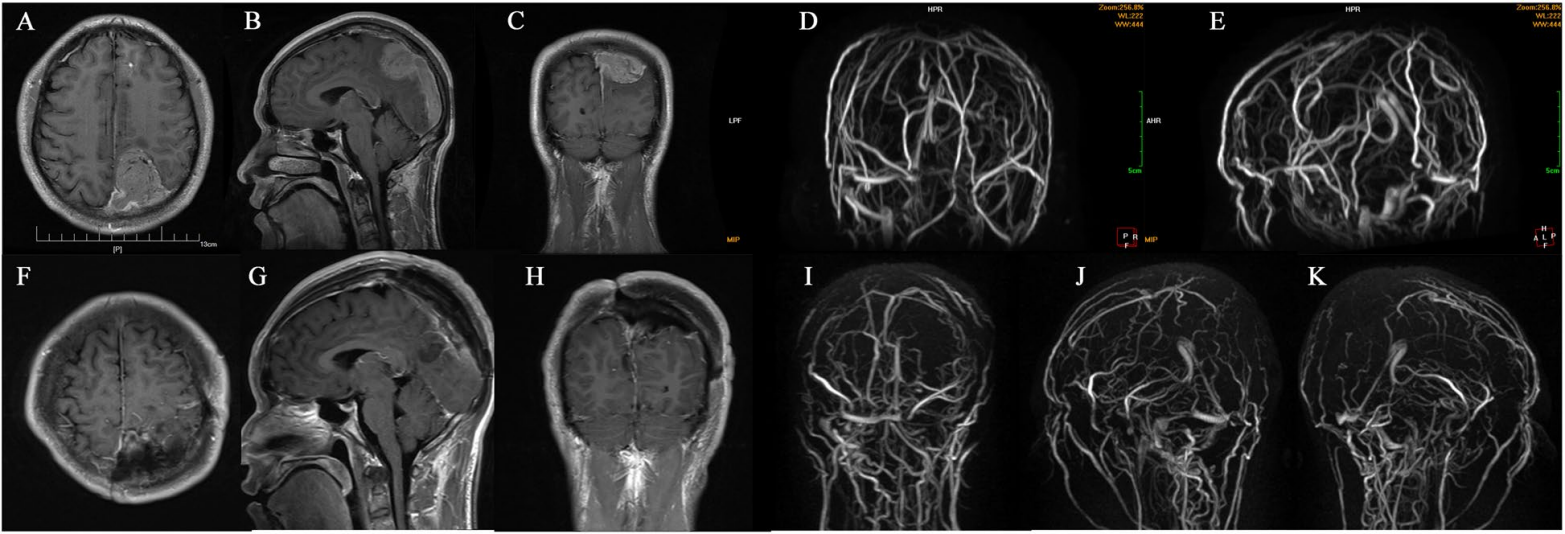
** A-E** patient with a type VI tumor was located in the posterior third of the superior sagittal sinus; the tumor filled the venous sinus and invaded the contralateral wall. **D** and **E** show the pre-operative MRV: the posterior third of the superior sagittal sinus was utterly occluded, surrounded by many compensated draining veins. **F-K** complete post-operative resection of the tumor: the tumor was resected together with the venous sinus, and no venous sinus reconstruction was performed. **I-K** post-operative MRV suggests the disappearance of the posterior third of the superior sagittal sinus, with surrounding compensatory vessels still present

**Fig. 7:**
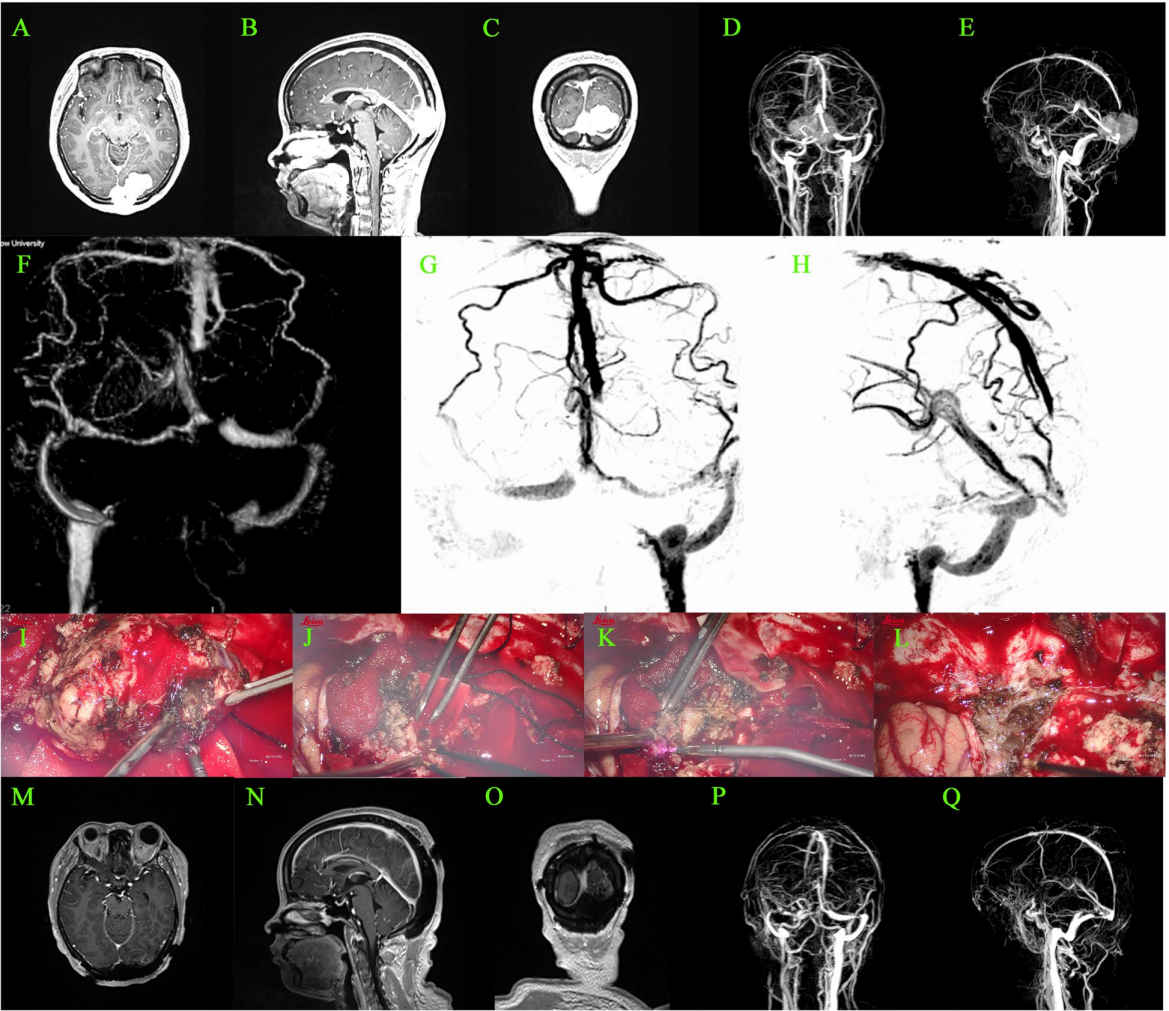
** A-C** type VI sinus confluence meningioma; **D-E** meningioma completely blocked the posterior third of the superior sagittal sinus, forming a collateral circulation; and the meningioma compressed the left transverse sinus abnormally thin; **F-H** pre-operative cerebral angiography, suggesting the absence of the posterior third of the superior sagittal sinus, with a small amount of compensated venous collateral drainage to the straight sinus; **I-L** intraoperative resection process of the tumor, removing the tumor together with the superior sagittal sinus, without sinus reconstruction; **M-O** complete post-operative resection of the meningioma; **P-Q** post-operative absence of the posterior third of the superior sagittal sinus, with a small amount of venous collateral circulation to the straight sinus

In all 68 cases, the extra-sinus mass was completely removed. Of these, 23 cases were identified as type I, wherein the outer layer of the sinus wall was excised and the inner layer was electrocoagulated (Simpson Grade II resection). Forty patients underwent complete resection of the tumor invading the venous sinus (Simpson Grade I resection), while the remaining 5 cases only had residual tissue electrocoagulated (Simpson Grade III resection). The approach for the surgical treatment was determined based on the sinus invasion classification (Fig. 3). In the case of type I, the tumor was completely stripped off from the venous sinus wall. For type II, the tumor tissue located on the lateral wall of the venous sinus was excised and then sutured (Fig. 4). In the 13 cases of type III (100%), the involved sinus wall was excised and sinus repair was performed in one stage. For type IV invasion, 12 (85.7%) underwent complete resection of the tumor and its invasion of the sinus wall, plus sinus wall repair (Fig. 5). The remaining two patients had either residual tissue treated with cautery electrocoagulation (the posterior third of the sagittal sinus) or complete resection with no venous sinus reconstruction (anterior third of the sagittal sinus). All five cases of type V invasion were treated by resectioning the invading sinus wall and restoring venous flow with a patch. For type VI venous sinus invasion, one case (20%) only had residual invading sinus wall treated with superficial cautery coagulation of the tumor tissue, while the remaining four cases (80%) underwent complete resection but no venous reconstruction (Figs. 6 and 7).

### Post-operative complication assessment

Four patients in this group of cases succumbed to death, resulting in an overall mortality rate of 4.4%. All fatalities were attributed to brain swelling caused by obstructed venous return and all four cases belonged to the type VI invasive sinus wall type, where only the sinus wall was removed but not reconstructed. Additionally, 10.3% of the patients experienced a worsening neurological deficit or complete loss of neurological function. Two of them (one type IV; one type V) underwent venous sinus wall repair, while the other five patients did not (type VI). No air embolism was reported after surgery.

**Fig. 8:**
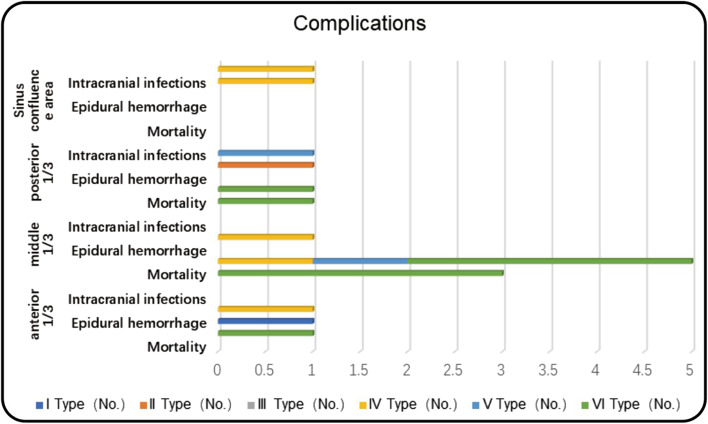
1 epidural hematoma: type I, tumor in the anterior third of the sagittal sinus, good prognosis; 3 patients with intratumoral hemorrhage: 1 type IV, sinus wall resection + patch repair, the anterior third of the superior sagittal sinus; 1 type IV, sinus wall resection + patch repair, the middle third of the superior sagittal sinus; 1 type II, sinus wall resection + sinus wall suture, posterior third of the superior sagittal sinus. 2 cases with good prognosis, the remaining 1 type IV, sinus wall resection + patch repair, middle third of the superior sagittal sinus, permanent neurological deficit. Two cases of intracranial infection: one type IV with sinus wall resection + patch repair, sinus confluence area; one type V with sinus wall resection + patch repair, located in the posterior third of the superior sagittal sinus. One case of subdural abscess: type IV with sinus resection without sinus reconstruction, located in the posterior third of the superior sagittal sinus. One patient with a subdural abscess: type IV, underwent sinus resection without sinus reconstruction, located in the posterior 1/3 of the superior sagittal sinus

Four patients developed post-operative intracranial hematomas: three cases of intraoperative hemorrhage and one epidural hematoma. After emergency cerebral hematoma removal, one patient with an epidural hematoma had a good prognosis; two of the three patients with intratumoral hemorrhage had a good prognosis, leaving one with permanent neurological deficits. In the anticoagulation subgroup, two patients had bleeding complications. Three patients developed intracranial infections (4.4%): two intracranial infections and one subdural abscess. The prognosis was good after the infections were treated with lumbar pool drainage and antibiotics corresponding to carbapenems, and after the subdural abscesses were treated with surgical clearance of the abscess, decompression of the debridement flap, and antibiotics (Fig. 8).

### Recurrence rate assessment

**Fig. 9:**
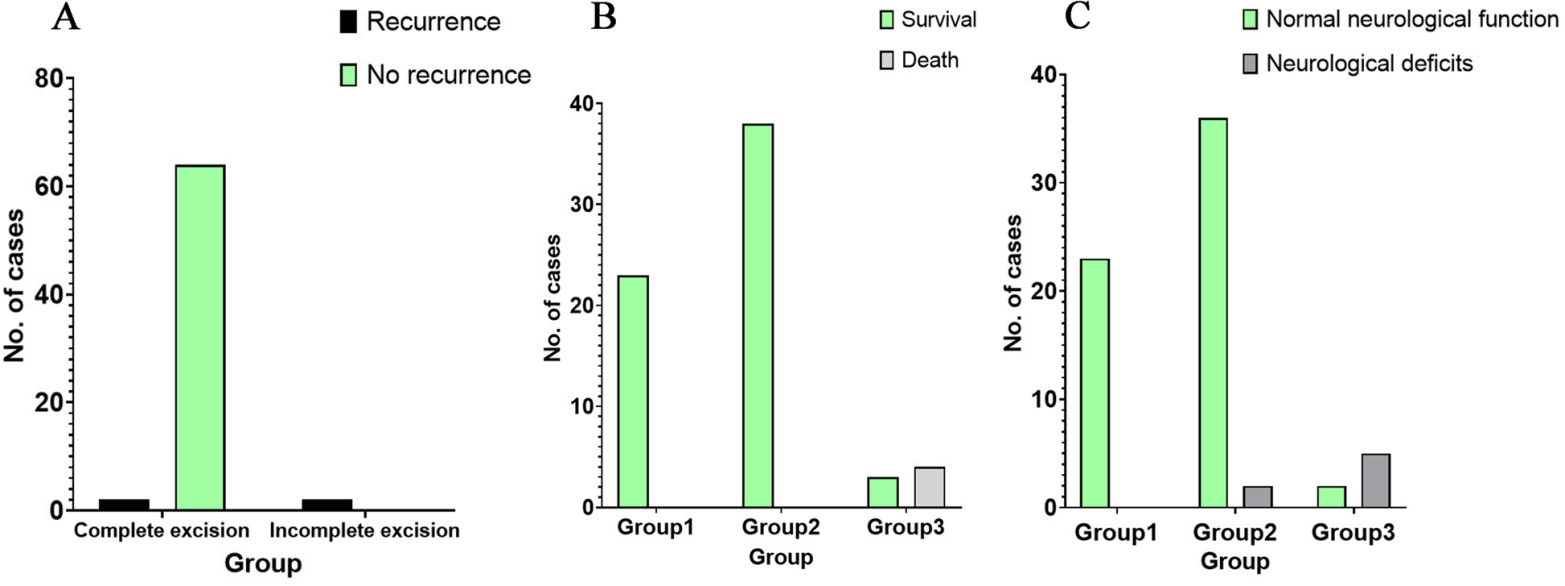
** A** The recurrence rate of the non-total resection group was higher than that of the total resection group; **B** The mortality rate of the third group was significantly higher than that of the remaining first and second groups; **C** The incidence of post-operative neurological deficits in the third group was considerably higher than that of the remaining groups

Four patients (5.9%) experienced tumor recurrence. One patient was located in the middle third of the superior sagittal sinus, one in the posterior third of the superior sagittal sinus, one in the sinus confluence area, and one patient was situated near the transverse sinus. The patient in the middle third of the superior sagittal sinus had type VI and only underwent extra-sinus tumor resection & electrocoagulation cautery of the sinus wall tumor without total resection, with atypical meningioma pathology. The patient in the posterior third of the sagittal sinus had type IV and had previously undergone meningioma resection at an outside hospital, and underwent extra-sinus tumor resection & electrocoagulation cautery at our hospital, with epithelial meningioma pathology that was not completely resected. The patient with transverse paranasal sinus had type IV and underwent infiltrative sinus wall resection and patch repair in our hospital, with atypical pathological findings. The patient with type V infiltrative sinus wall type, located in the sinus confluence area, underwent infiltrative wall resection & sinus wall patch repair in our hospital, with post-operative pathology suggesting transitional meningioma. The recurrence rate (Fig. 9) was higher in the non-total resection patients than in the complete resection group (*P* = 0.0026).

### Magnetic resonance venography assessment

All 38 patients who underwent venous reconstruction (Type II: 8; Type III: 13; Type IV: 12; Type V: 5) had MRV data available pre-and post-operatively, within two weeks of the procedure. Of the 8 Type II cases, all had patent venous sinuses post-operatively. Of the 30 repair cases, 24 (80%) were patent, with 12 (92.3%) Type III invasive types having post-operative MRV suggestive of patency. Additionally, 10 (71.4%) Type IV patients with sinus wall infiltration had MRV patency, as did 3 (60%) Type V cases.

### Karnofsky performance status scale assessment

In type I to type III cases, there was a slight decrease in KPS from preoperative levels, with no significant improvement in KPS after surgery (*P* = 0.67), likely due to the milder preoperative symptoms and higher KPS. For type IV patients, the 12 patients who underwent vein reconstruction experienced a decrease in KPS from 90.67 ± 0.69 to 86.67 ± 2.8, with no significant statistical difference, likely due to the small sample size. For type V, there was no significant deterioration in patients after surgery, with a KPS score of 88.8 ± 1.53 and 83.2 ± 3.9 (*P* = 0.06). However, in type VI patients, all of whom did not undergo venous reconstruction, the post-operative KPS score was significantly worse: 59 ± 6.9 compared to 10 ± 10 (*P* = 0.00317, t-test) (Table 3).

**Table 3:** Pre-operative and post-operative KPS scores for different surgical approaches

Type of Invasion & Surgical Strategy (no. of cases)	KPS Score			*P* value
	Preop	Postop	Δ	
Type I				
Peeling (23)	94.3 ± 0.25	94.22 ± 0.27	0.1	0.84
Type II				
Reconstruction (8)	92.88 ± 0.4	92.38 ± 0.63	0.5	0.49
Type III				
Reconstruction (13)	90.85 ± 0.98	90.15 ± 1.44	0.7	0.34
Type IV				
Reconstruction (12)	90.67 ± 0.69	86.67 ± 2.8	4	0.086
No reconstruction (2)	91.50 ± 0.5	82.5 ± 2.5	9	0.33
Type V				
Reconstruction (5)	88.8 ± 1.53	83.2 ± 3.9	5.6	0.06
Type VI				
No reconstruction (5)	59 ± 6.9	10 ± 10	49	0.00317*

* means that the *P* value is less than 0.05, which is statistically significant

### Subgroup analysis

**Fig. 10:**
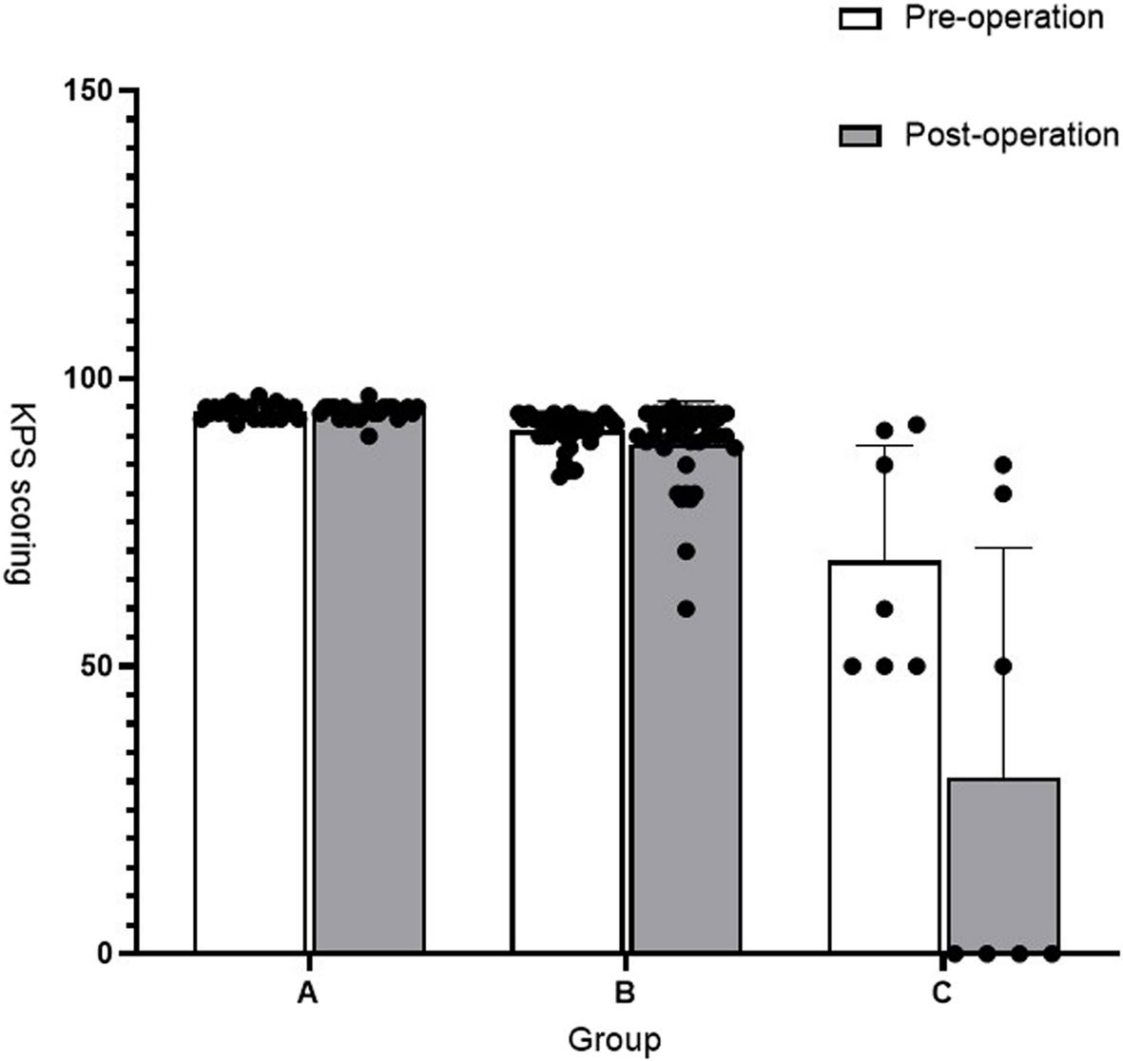
The results suggest that patients in group 3 who did not undergo venous reconstruction showed significant post-operative deterioration

The study population was divided into three groups: Group I included type I cases (23 cases); Group II comprised type II to type VI patients who underwent one-stage venous suture or patch repair (38 cases); and Group III was composed of type II to type VI cases without venous reconstruction (7 cases). The mortality rate in Group III was significantly higher than in the remaining two groups (*P* < 0.0001). Furthermore, the incidence of neurological deficits or complete loss of neurological function in the third group was significantly higher than in the other two groups (*P* < 0.0001). Analysis of the pre-and post-operative KPS scores revealed that Group III patients who did not undergo venous reconstruction experienced a significant post-operative deterioration (*P* = 0.016) (Fig. 10).

## Discussion

Surgical intervention for paravebous sinus meningiomas that have spread to major venous sinuses is a difficult decision. There is disagreement over whether to take a more aggressive approach to remove the portion of the tumor that has invaded the venous sinuses and the sinuses themselves; a remaining tumor may result in recurrence, yet a thorough resection may cause venous sinus infarction. Therefore, we must find a compromise between the amount of tumor resection and the risk of venous complications.

The most common cause of meningioma recurrence is failure to achieve radical resection. The Simpson classification is widely accepted as the best indicator of meningioma recurrence, with a recurrence rate of 6%, 16%, 29%, and 37–85% for grades I-IV, respectively. DiMeco et al. have demonstrated a statistically significant difference in 5- and 10-year recurrence rates among patients with Simpson grade I, II, and IV resections, thus emphasizing the importance of achieving a radical resection as the primary treatment for meningioma [11]. Several studies have focused on the effects of radiation therapy on paraneoplastic meningiomas of the venous sinus, with the results suggesting that those with smaller tumors may be able to benefit from this form of treatment [12–14]. Despite the fact that radiotherapy has been shown to not reduce the recurrence rate of incompletely resected meningiomas, its efficacy for post-operative residual paranasal meningiomas is still uncertain [15]. Our study demonstrated that complete resection of the tumor was achieved in 97.1% (66/68) of cases, with a follow-up period of 33 to 57 months. The recurrence rate was 5.9% (4/68), with two of the four patients with recurrence not having undergone complete resection. The recurrence rate was significantly higher in the group without complete resection than in the group with complete resection (P = 0.0026, Fisher’s test). Therefore, we strongly recommend aggressive complete resection of paraneoplastic sinus meningiomas to reduce recurrence.

There is still debate as to whether venous sinus reconstruction is necessary. Research has indicated that the removal of the anterior third of the superior sagittal sinus, along with the tumor, does not necessitate venous reconstruction and does not have severe repercussions [16, 17]. Professor Enrico believes that active tumor resection can achieve a higher tumor control rate, while active tumor resection without reconstruction of the venous sinus will bring higher venous complications and deterioration of original dyskinesia [18]. Professor N.Desse and Qazi Zeeshan believe that radical resection of parasinus meningioma and reconstruction of the venous sinus is the best treatment for long-term tumor control [19, 20].In a study conducted by Sindou et al., which included 100 patients with paraganglioma of the superior sagittal sinus, it was found that aggressive resection of the middle and posterior thirds of the sinus could potentially lead to an increased rate of complications. Venous sinus reconstruction was performed in 45 of the patients whose tumors had invaded the sinus wall or cavity, resulting in 3% of the patients dying due to brain swelling, 8 other patients developing permanent neurological deficits, and 4 patients developing recurrence at a mean follow-up of 8 years, two of whom had WHO grade II meningiomas. It was concluded that reconstruction of the venous sinus is necessary after radical tumor surgery and that there is a potential role for the venous sinus in cases of complete occlusion, as often compensatory collateral circulation is insufficient to fully compensate for the venous sinus reflux effect [8]. DiMeco et al. conducted a study of 108 individuals with meningioma invasion of the sagittal sinus, and their results suggested radical resection with in situ suture repair for partial sinus invasion. However, for total occlusion, complete sinus resection was recommended, which lead to severe post-operative brain swelling in 9 (8.3%) patients and post-operative hemorrhage in 2 patients (1.85%) [11]. Mantovani et al. aimed to achieve complete tumor resection while preserving venous circulation. To restore venous blood flow, they employed primary suturing or repair with a dura or patch, ensuring at least one-third of the dural wall of the venous sinus remained intact. Additionally, they recommended post-operative antiplatelet therapy, which often permitted some degree of blood leakage. The results of their study on 21 patients revealed that 85.7% of the venous sinuses were either patent or narrow but patent. Three patients experienced venous sinus occlusion, however, no post-operative complications such as cerebral edema, venous infarction, or hematoma occurred due to the presence of compensatory venous circulation. This high rate of post-operative venous sinus patency indicates that the use of antiplatelet therapy and autologous material repair is a viable approach [21].

Nevertheless, not all results are in agreement with the progressive management of the venomous sinus and the venomous sinus. Caroli et al. conducted a study on 328 patients with invasive meningiomas of the parasagittal sinus, of which 215 had a complete resection of the superior sagittal sinus and 113 had a partial resection. In the case of total occlusion, complete excision of the involved venous sinus was performed, whereas, in the case of partial occlusion, partial excision was followed by radiotherapy. This method resulted in ten patients developing post-operative dyskinesia. This treatment approach by Caroli did not seem to lessen post-operative complications [22].

The present article outlines the surgical strategy adopted in the management of paravebous sinus meningiomas involving major venous sinuses. To reduce the likelihood of post-operative recurrence, the intraoperative resection of the tumor and the invaded sinus wall is necessary. Wherever possible, sinus walls with minor defects are repaired with suture techniques, while more substantial defects require artificial material for repair. In this study, sinus reconstruction was attempted in 84.4% of the 45 cases with sinus wall and sinus cavity invasion. The pre-and post-operative KPS scores were not significantly different in patients of types I to V, however, those with type VI (who did not receive venous reconstruction) had significantly worse post-operative KPS scores. In the subgroup analysis, four patients died in group III, with the mortality rate significantly higher than in the other two groups. Additionally, seven patients experienced worsening symptoms of neurological deficits or complete loss of neurological function, including two patients in group II and five in group III. Consequently, it is suggested that venous sinus repair is necessary, and the use of MRV and multimodal fusion technology is essential for paravebous sinus meningiomas. This technology is used pre-operatively to simulate the position of the tumor in relation to the venous sinuses, draining veins, and surrounding functional brain structures, to evaluate the extent of tumor invasion of the venous sinuses, and to determine the surgical strategy. Post-operatively, it is used to assess the patency of the venous sinuses.

We are of the opinion that a comprehensive tumor resection with the aid of imaging multimodality techniques and post-operative anticoagulation can reduce post-operative complications and reduce recurrence rates. However, due to the lack of patients who underwent venous sinus resection and autologous vein graft in this study, we could only resort to venous sinus sutures or patch repairs. To confirm the positive efficacy of total tumor resection and venous sinus reconstruction, we will need to expand the sample size.

## Conclusion

The results of this study suggest the necessity of a complete resection of the tumor, including the invasive venous sinus component, as the recurrence rate was found to be relatively low at 5.9%. Moreover, patients who did not undergo venous reconstruction showed a significant deterioration in their clinical condition compared to other subgroups, thus highlighting the importance of venous sinus reconstruction.

## Supplementary Information

**Additional file 1.** Patient's raw data.

## Data Availability

The raw data for this study have been uploaded to the supplementary material, and these data are available from the corresponding authors upon reasonable request.
